# 

**Published:** 1906-02-15

**Authors:** 


### ANNOUNCEMENTS.

THE QUONEHTACUT CLUB, of Hartford, Connecticut, has changed its name to the HORACE WELLS CLUB. Dr. Wells surely did not live a useless life and he should be again memorialized for being the indirect cause of consigning to oblivion such an unpronounceable and almost unprintable name. Dr. J. H. McManus is the president, and there ought to be comething doing in that society this year.

DR. M. H. CHAPPELL, one of the best known dentists in Southeastern Indiana died of apoplexy, December 15, 1905, at the age of 64, in Knightstown, his home town where he had practiced for forty years.

FACULTY RESIGNATION.—Drs. Thorpe, Le Cron, Suma and six other members of the faculty of the Dental Department of Barnes Medical University, of St. Louis, have resigned their positions as teachers in that school. The reasons given are that the trustees are not ready to comply with the requirements of the Missouri State Board of Dental Examiners, and of the National Board of Examiners, as to faculties, etc.

DR. FRANKLIN S. GRAVES, 71 years of age and one of the oldest practitioners of dentistry in Michigan, died at his home in Battle Creek, December 16th, 1905, from paralysis. His son Dr. F. P. Graves has carried on the practice for several years.

---

MARRIED.—Dr. George M. Freeman, of Seattle, Washington, was married December 25th, 1905, to Miss Geneva Violet Long, of Walla Walla.—Dr. Sidney G. Main, of Ishpeming, Michigan, was married to Miss Maude Ethel Moore, of Valparaiso, Indiana, December 28th, 1905. The REGISTER extends cordial greetings to both couples.

---

OHIO DENTAL LAW CONSTITUTIONAL.—December 3rd, 1905, the Supreme Court of Ohio declared the State Law constitutional in a suit of Elsworth Glenn, *v.s.* the State of Ohio. Glenn was indicted by the Franklin County Grand Jury in 1902. Glenn then applied for a license under the former law, but the board declined to issue him a license. Glenn then began mandamus proceedings to compel the board to issue him a license, but he was defeated in two lower courts. The case was then carried to the Supreme Court and the board's action was sustained. This establishes the constitutionality of the law.

